# Recent Advances in Applications of Ionic Liquids in Miniaturized Microextraction Techniques

**DOI:** 10.3390/molecules23061437

**Published:** 2018-06-13

**Authors:** Maria Kissoudi, Victoria Samanidou

**Affiliations:** Laboratory of Analytical Chemistry, Department of Chemistry, Aristotle University of Thessaloniki, 541 24 Thessaloniki, Greece; marikiss@chem.auth.gr

**Keywords:** ionic liquids, sample preparation, microextraction, solid-phase microextraction, dispersive liquid-liquid microextraction, single-drop microextraction, stir bar sorptive extraction, stir cake sorptive extraction

## Abstract

Green sample preparation is one of the most challenging aspects in green analytical chemistry. In this framework, miniaturized microextraction techniques have been developed and are widely performed due to their numerous positive features such as simplicity, limited need for organic solvents, instrumentation of low cost and short time of extraction. Also, ionic liquids (ILs) have unequivocally a “green” character, which they owe to their unique properties including the re-usage, the high reaction efficiency and selectivity in room temperature, the ability to dissolve both organic and inorganic compounds, and thermal stability. In the present review, the recent advances in the application of ionic liquids in miniaturized liquid and solid phase extraction techniques as extractants, intermediate solvents, mediators and desorption solvents are discussed, quoting the advantages and drawbacks of each individual technique. Some of the most important sample preparation techniques covered include solid-phase microextraction (SPME), dispersive liquid-liquid microextraction (DLLME), single-drop microextraction (SDME), stir bar sorptive extraction (SBSE), and stir cake sorptive extraction (SCSE).

## 1. Introduction

Nowadays, sample preparation procedures are more than ever linked with the protection of the environment following the philosophy of Green Analytical Chemistry (GAC). A typical analytical procedure consists of three main parts: sampling, sample preparation and final analysis. It is generally known that almost the 75% of its time is spent in the stage of preparation, so it is a critical part of the analytical procedure. According to GAC, new environmentally friendly instrumentation and methodologies with the minimum emissions of pollutants and environmental sustainability in terms of cost and energy of chemical laboratories are the major aims in the field of sample preparation [1,2,3].

Among the sample preparation methods used to clean up and concentrate analytes, liquid-liquid extraction (LLE) and solid-phase extraction (SPE) are the most famous and widely used. Despite their universal application, these methods are accompanied with some drawbacks, such as time-consumption, high cost, inability to extract polar compounds (mainly for LLE), use and disposal of great amount of toxic solvents, complication to automate and potential evaporation and dissolution in a proper solvent prior the analysis, which adds an extra step in the whole procedure. Miniaturization in sample preparation techniques is the key to overcome these above drawbacks. Furthermore, the combination of ionic liquids with the miniaturized sample preparation techniques could be the panacea for the pre-stated limitations [4,5].

Ionic liquids (ILs) are organic molten salts with melting point lower than 100 °C in contrast with the common molten salts (fused salts), such as sodium chloride which becomes liquid at 801 °C. “Room temperature ionic liquids” (RTILs) is a term which describes a group of ionic liquids with melting points at or below room temperature (25 °C). ILs are composed of positive and negative ions, usually a bulky organic nitrogen-containing cation, such as imidazolium, pyridinium, pyrrolidinium, phosphonium or ammonium, and a halogen-based organic or inorganic anion, including trifluoromethylsulfonate [CF_3_SO_3_]^−^, bis[(trifluoromethyl)sulfonyl]imide [(CF_3_SO_2_)_2_N]^−^ (i.e., NTf_2_), trifluoroethanoate [CF_3_CO_2_]^−^ or Cl^−^, PF_6_^−^, BF_4_^−^, respectively, whereas water and other organic solvents are composed of molecules [6,7]. The first discovered RTIL is the ethylammonium nitrate [EtNH_3_][NO_3_] with melting point at 12 °C by Walden in 1914 [8]. However, remarkable progress has been achieved in the field of ionic liquids since 1992, when Wilkes and Zaworotko introduced a promiscuous generation of ionic liquids, the air and water stable 1-ethyl-3-methylimidazolium-based ILs, [EtMeim]BF_4_ and [EtMeim]MeCO_2_ [9]. The future of ILs belongs to the deep eutectic solvents (DESs), an interesting subclass of ILs which are less toxic, they have a stronger ecofriendly profile and they can be produced easier and cheaper than the ILs. One of their main drawbacks is their solid state in room temperature and their high viscosity [10].

**Figure 1:**
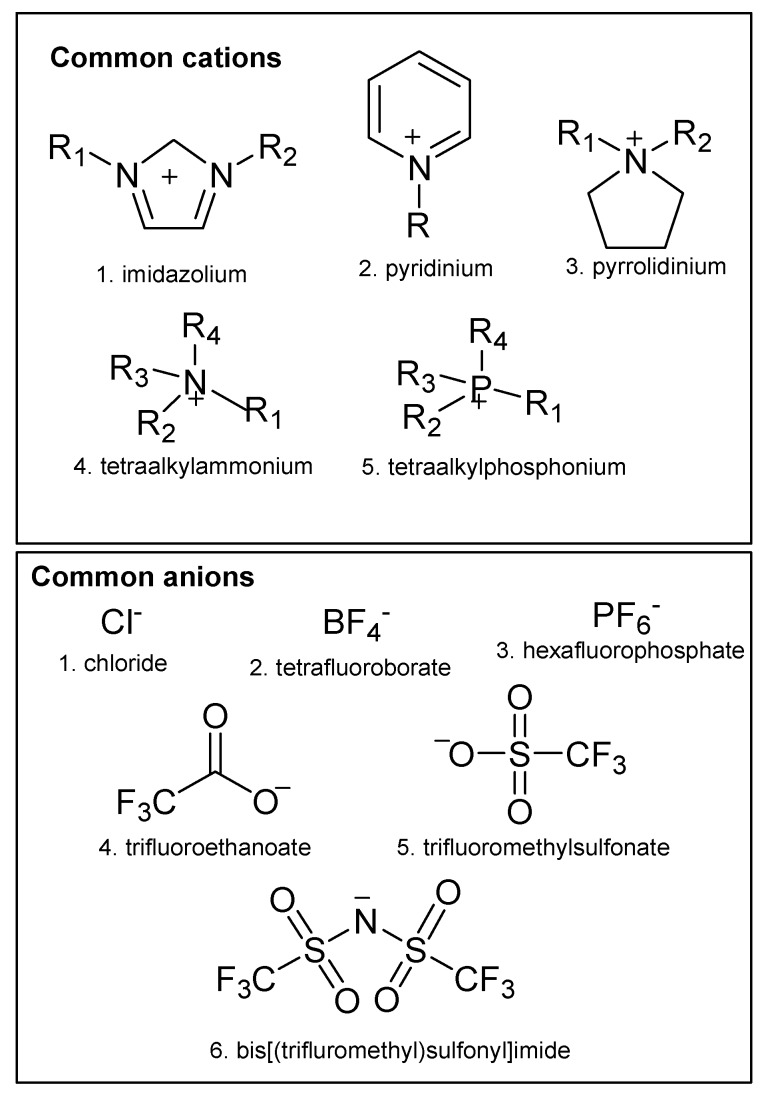
Structures of common cations and anions of ionic liquids.

The structures of the most commonly used ILs are represented in Figure 1. ILs owe the fact that they remain liquid in temperatures below 100 °C to the large size of the ions that they consist of, avoiding the packing of ionic lattice, which happens in inorganic salts. Compared with conventional solvents, ILs have some special physicochemical properties, such as low volatility, high electrical conductivity, long-term thermal and chemical stability, low flammability, and low vapor pressure. The negligible vapor pressure is one of the properties that makes ILs “green solvents”, as they contribute to the reduction of atmospheric pollution and the health risks that are entailed. The chemical and physical properties of ILs, such as polarity, hydrophobicity, viscosity, depend on the anionic and cationic constituents giving them the characterization, “designer solvents” [11,12,13,14,15].

Among the approximate 10^18^ anion-cation combinations that can synthesize ionic liquids, there is a great majority of combinations that have toxic effects on water, environment, bacteria, plants, fish and human [16]. In a recent quantitative structure-property/activity relationship study of Zhao et al., a database for the toxicity of ILs has been established [17]. The authors conclude that the toxicity of ILs is related to the number of oxygen atoms that are present in the molecule. Also, the anion [NTf_2_]^−^ influences the level of toxicity of ILs. However, the relationship of the toxicity and the length of the alkyl side chains is not always proportional. Therefore, it is crucial researchers get advice from relevant databases before synthesis and use of ILs either for industrial or laboratory use. Furthermore, there are guides not only for the selection of ionic liquids but for the solvents generally. One of the most useful tools for solvent selection has been proposed by scientists of Pfizer and it is presented in Table 1 [18]. ILs are not always “green” solvents. Some of them are made from toxic ions and they are not biodegradable, despite their promiscuous physicochemical properties. Of course, not all the organic solvents that are used in the laboratories are toxic and harmful for the environment, but some of them can be replaced by other types of solvents with lower environmental impact.

**Figure 2:**
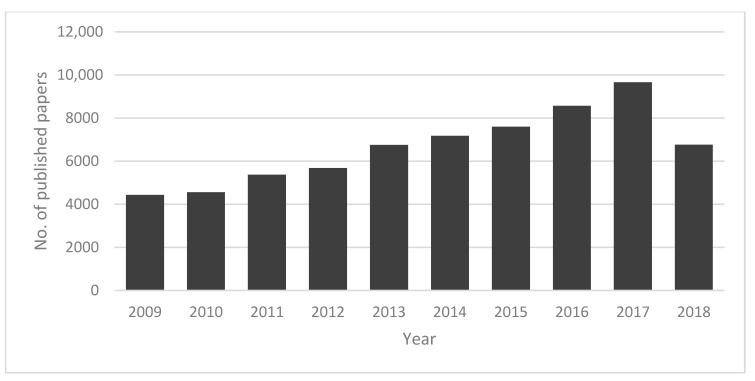
Number of published scientific papers worldwide per year (from 2009 to 2018) on the applications of ionic liquids in sample preparation techniques (based on Scopus and ScienceDirect).

This review summarizes the recent advances in application of ionic liquids in miniaturized liquid and solid phase extraction techniques as extractants, intermediate solvents, mediators, and desorption solvents. As it is shown in Figure 2, the amount of published studies associated with ionic liquids has grown rapidly within the last decade. Some of the most important sample preparation techniques covered include solid-phase microextraction (SPME), dispersive liquid-liquid microextraction (DLLME), single-drop microextraction (SDME), stir bar sorptive extraction (SBSE), stir cake sorptive extraction (SCSE).

## 2. Ionic Liquids in Miniaturized Microextraction Techniques

### 2.1. Solid-Phase Microextraction (SPME)

Solid-phase microextraction (SPME) is a widely known sample preparation technique, combining sampling and pre-concentration, due to its quickness and cost-effectiveness with absence of organic solvents. Since its establishment by Pawliszyn and co-workers it has been performed combined with several types of commercial sorbent coatings, such as polydimethylsiloxane (PDMS), polyacrylate, carboxen, PDMS-carboxen or divinylbenzene (DVB) depending on the analyte [19,20].

In a recent study of Tang and Duan, a porous polymeric ionic liquid, poly(1-vinyl-3-(4-vinyl-benzyl)imidazolium chloride), was synthesized and used as a sorbent coating for SPME for the analysis of polar organic acids [21]. The results showed that the fiber was more sensitive and practical for the extraction of polar compounds in comparison with the commercial fibers.

Ionic liquids compromise another effective type of sorbent coating that can be coupled with SPME technique in two different modes, headspace (HS-SPME) and direct immersion (DI-SPME). In the first mode, the sorbent coating is exposed to the headspace of the sample where the target analyte is present, while in the second the SPME-fiber is pushed out of the hollow needle and immersed into the sample directly. Following the sorption of the analyte in both cases, the fiber is drawn into the needle, the needle is withdrawn from the sample vial and transferred to the injection port of an analytical instrument, where desorption of the analyte takes place and the analysis is carried out [4,22].

**Figure 3:**
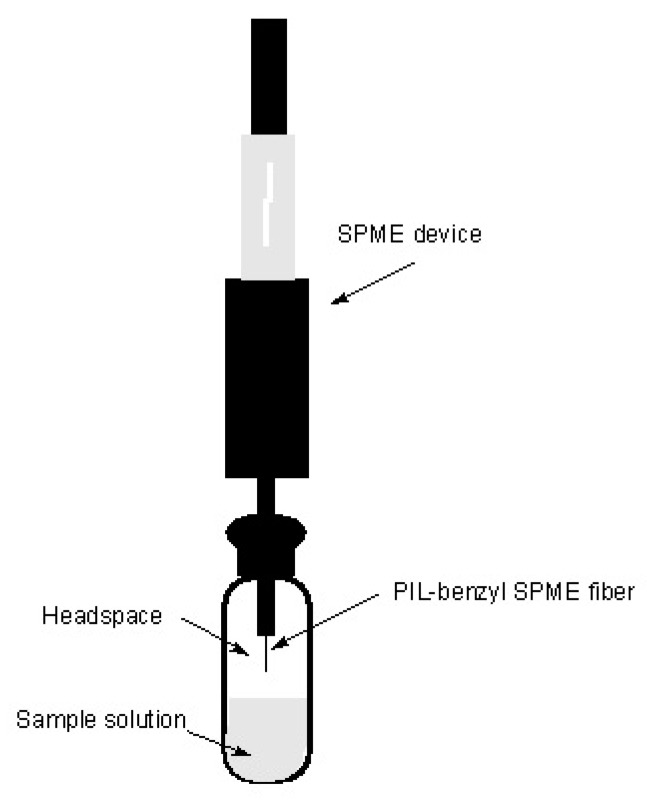
Headspace-solid phase microextraction procedure for the extraction of PAHs in water sample using a polymeric ionic liquid-based fiber.

A benzyl-functionalized crosslinked polymeric ionic liquid (PIL) was developed by Merdivan et al. and successfully used as a sorbent coating in headspace solid-phase microextraction (HS-SPME) coupled to gas chromatography with flame-ionization detection for the determination of seven volatile polycyclic aromatic hydrocarbons (PAHs) in environmental water samples [23]. The VBHDIM-NTf_2_ IL monomer and (DVBIM)_2_C_12_-2NTf_2_ IL cross-linker were used for the synthesis of the crosslinked PIL-based sorbent coating. Figure 3 represents the schematic illustration of HS-SPME procedure using the benzyl-PIL fiber for the extraction of PAHs. Compared to the commercial PDMS fiber coating for the PAHs, the crosslinked PIL fiber showed higher log K_fs_ due to the presence of benzyl moieties within the IL monomer and IL crosslinker of the PIL sorbent coating indicating its superior affinity, higher sensitivity with low LODs, ranged from 0.01 to 0.04 µg L^−1^ for the PIL–benzyl fiber and from 0.01 to 0.07 µg L^−1^ for the PDMS fiber. The linearity, the RSD values and the relative recovery values were more satisfactory for the PIL–benzyl fiber than the PDMS fiber. The presence of benzyl moieties in the PIL coating material enhances π-π interactions between the target compounds (PAHs), which are composed of aromatic rings, and the sorbent coating.

In-tube SPME is an improved mode of SPME sample preparation technique introduced by Pawliszyn in 1997, where the coupling of SPME with HPLC is achieved more conveniently. Sun et al. developed a fiber-in-tube solid phase microextraction device with copper support modified via chemical bonding with ionic liquids. Especially, one copper tube was filled with eleven copper wires functionalized with ionic liquids and it was combined online with a high-performance liquid chromatography system to strengthen extraction capacity and eliminate dead volume, building an online in-tube SPME-HPLC system. In this framework, an in-tube SPME-HPLC method for the determination of estrogens in water samples was developed with high enrichment factors and satisfactory sensitivity (LODs: 0.02–0.05 g L^−1^). The presence of ionic liquids in the metal support was crucial for the effectiveness of the developed method increasing the stability of in-tube SPME device and improving the sensitivity by increasing sample volume with a high sampling rate [24].

### 2.2. Dispersive Liquid-Liquid Microextraction (DLLME)

Dispersive liquid-liquid microextraction (DLLME) was first introduced by Rezaee et al. in 2006 as a novel alternative technique for the extraction and preconcentration of organic compounds in water samples [25]. The fundamentals of DLLME technique are based on the mixing of an aqueous sample containing the analytes with a non-miscible with water organic solvent, used as an extractant, together with a small amount of a dispersive solvent, which is miscible in both water and the extractant solvent. The mixing of the two solvents should be preceded their injection into the sample by a syringe or a micropipette. Gentle manual shaking of the mixture disperses the organic extractant as fine droplets to form a homogenous cloudy solution in which partition of the analytes takes place. Then, the sample is centrifuged, and the sediment phase is collected and determined with the appropriate analytical method [26].

Since its introduction, DLLME has been performed as an extraction technique in various fields of chemistry, such as analytical, environmental, biochemistry, medicinal, pharmaceutical, toxicology and many others. Its increasing popularity is owed to some significant properties, such as the simplicity, the low cost, and the environmentally friendly profile. Furthermore, the dispersive mode improves the extraction kinetics by increasing the contact surface between the extractant and the sample. The most critical part of this technique is the choice of the extraction solvent, which should have a density greater than water’s density and to form a cloudy solution with the dispersive solvent [27,28].

Taking into consideration the properties of ionic liquids as extraction solvents described before, the combination of ILs in DLLME process could be characterized promiscuous. Zhou et al. and Baghdadi and Shemirani were the first researchers who performed the first successful applications of ILs in DLLME process, namely IL-DLLME, for the determination of organophosphorus pesticides in environmental samples and for the extraction of mercury from water samples, respectively [29]. While, Liu and coworkers introduced the conventional IL-DLLME combined with HPLC-DAD for the determination of heterocyclic insecticides in water using the IL 1-hexyl-3-methylimidazolium hexafluorophosphate, ([C_6_MIM][PF_6_]), as the extractant and methanol as the dispersive solvent [30].

### 2.3. Stir Bar Sorptive Extraction (SBSE)

Stir bar sorptive extraction (SBSE) was initially introduced in 1999 by Baltussen and co-workers [31]. It uses a stir bar for the extraction consisting of a magnet covered with glass, which in turn is coated by a layer (typically 0.5–1 mm) of sorptive material, usually polydimethylsiloxane (PDMS). The bar is subsequently inserted into a vial, which contains the aqueous sample and it is stirred until equilibrium of analytes concentration between sorbent and sample matrix is reached. After the extraction, the bar is removed and transferred to a clean vial, where the target compounds are analyzed by liquid or gas chromatography by liquid or thermal desorption [32].

Fan and co-workers introduced a novel approach for the extraction and determination of nonsteroidal anti-inflammatory drugs (NSAIDs) by high-performance liquid chromatography-ultraviolet detection (HPLC-UV) [33]. The researchers synthesized an ionic liquid, 1-allylimidazolium tetrafluoroborate ([AIM][BF_4_]) chemically bonded sol-gel coating for stir bar sorptive extraction using γ-(methacryloxypropyl)trimethoxysilane (KH-570) as a bridging agent, which showed excellent mechanical strength and chemical/thermal stability compared to the conventional PDMS-based or C18 coating materials. After the optimization of the critical parameters of the technique, such as stirring rate, extraction time, desorption solvent, pH, salt effect, the developed method showed satisfactory reproducibility with RSDs lower than 7.6% and high sensitivity (LODs: 0.23–0.31 μg L^−1^) for the determination of three NSAIDs. The proposed method is applicable in environmental, food and biological samples

In the framework of the improvement and development of microextraction techniques, the combination of two or more techniques could induce significant enhancements to the extraction procedure reducing the extraction time, the cost, or the sensitivity. Two microextraction techniques that can be easily conflated is the stir bar sorptive extraction (SBSE) and dispersive liquid-liquid microextraction (DLLME) introducing the stir bar dispersive liquid microextraction (SBDME). In this approach, Chisvert et al. used a magnetic ionic liquid (MIL) and a neodymium-core magnetic stir bar as the extraction phase [34]. At low stirring rates it acts similar to SBSE while the MIL remains in the stir bar surface. At higher stirring speed the MIL disperses into the solution acting similar to DLLME. When the extraction is over, the stirring stops and the MIL retrieves onto the stir bar surface due to its magnetic properties. The desorption of the MIL-coated stir bar contained the preconcentrated analytes performed thermally in a gas chromatography-mass spectrometry (TD-GC-MS) system. The above approach was applied for the extraction of lipophilic organic UV filters from aqueous environmental water samples.

In a recent work by Benedé et al. another successful combination of stir bar sorptive extraction (SBSE) and dispersive liquid-liquid microextraction (DLLME) has been achieved and the stir bar dispersive liquid microextraction (SBDLME) was created for the determination of ten polycyclic aromatic hydrocarbons (PAHs) in natural water samples [35]. A neodymium stir bar magnetically coated with a magnetic ionic liquid (MIL), the [P_6,6,6,14_^+^][Ni(II)(hfacac)_3_^−^], was used as extraction device. After the stirring, the MIL is magnetically retrieved onto the stir bar and then subjected to thermal desorption-gas chromatography-mass spectrometry system (TD-GC-MS). In contrast to other methods for the determination of PAHs, this one requires less time and manipulation for the sample preparation, solvent evaporation is not a mandatory step and the sensitivity level is more than satisfactory allowing the determination of PAHs in aqueous sample at the low ng L^−1^ levels. 

### 2.4. Single-Drop Microextraction (SDME)

Single-drop microextraction technique (SDME) was first introduced by Liu and Dasgupta in 1995 as an alternative extraction process eliminating the problems of solvent evaporation, which exists in LLE and SPE, and the degradation of SPME fiber [36]. The basis of this technique is the distribution of the target compounds and a microdrop of solvent that is suspended in the tip of a microsyringe needle. It uses small volumes of organic solvents and simple equipment reducing the total cost of each individual application. SDME can be performed with direct immersion (DI-SDME) in the aqueous sample or in the headspace of the sample (HS-SDME). The main limitation of this technique is the fact that the stability of droplet is highly depended on the used solvent. Thus, solvents with low viscosity, high vapor pressure and low surface tension decrease the effectiveness of the extraction process. ILs could be used as an alternative to the common organic solvents used in SDME.

Jiang et al. used the SDME technique for the extraction and concentration of flavor and fragrance substances in fruit juices [37]. A hydrophilic IL, 1-hexyl-3-methylimidazolium tetrafluroborate, used successfully as an extraction solvent. The method was validated after the optimization of the main parameters, such as the volume of solvent microdrop, the extraction time, the enrichment time, the temperature, the pH of the sample solution, the height of the microdrop above the solution surface. The method demonstrates satisfactory sensitivity and accuracy with relative standard deviations lower than 9.1%.

A headspace single-drop microextraction (HS-SDME) method for the determination of aromatic analytes by HPLC was developed by An et al. using as solvents tetrachloromanganate ([MnCl_4_^2−^])-based magnetic ionic liquids [38]. A rod magnet was used to sustain the microdroplet of MIL during HS-SDME. High stability of the microdroplet under high temperature and long extraction times was achieved due to the magnetic susceptibility of the MILs. The method showed high sensitivity and precision for the target compounds, and satisfactory relative recoveries from an application in real sample.

He and co-workers developed an ionic liquid-based headspace single drop microextraction combined with high-performance liquid chromatography (HS-SDME-HPLC) method for the determination of camphor and trans-anethole in licorice tablets [39]. The ionic liquid used as the extracting medium was 1-bityl-3-methylimidazole hexafluorophosphate. The method showed satisfactory stability and sensitivity after setting the volume of the IL microdrop to 12 μL. The method is simple, rapid, selective, precise, accurate and linear for the target compounds. SDME technique has the advantage that allows the single step separation, purification and enrichment improving the signal-to-noise ratio and ensuring the accuracy of the method as there was no loss of volatile components. The method is expected to be widely performed for the preparation of volatile compounds of drugs with high boiling points.

### 2.5. Stir-Cake Sorptive Extraction (SCSE)

Stir-cake sorptive extraction technique is an improved version of SBSE introduced in 2011 [40]. The SCSE device consists of a holder made of iron where the stationary phase is placed. The most common used extractive mediums in SCSE are monolithic cakes designed and prepared properly according to the extracted analytes. In the literature the most often used extraction phases are poly(4-vinylbenzoic acid-divinylbenzene) (VBADB) sorbents based on polymeric ionic liquids.

Wang et al. proposed a new SCSE approach using as sorbent a polymeric ionic liquid monolith obtained by the in situ copolymerization of an ionic liquid, 1-allyl-3-methylimidazolium bis[(trifluoro methyl)sulfonyl]imide (AMII) and divinylbenzene (DB) in the presence of *N*,*N*-dimethylformamide [41]. By coupling SCSE–AMIIDB with high performance liquid chromatography/diode array detection (SCSE–AMIIDB–HPLC/DAD) a simple and effective method for the determination of trace benzimidazoles (Bas) residues in water, milk and honey samples was established with low LODs and high levels of recovery. The SCSE-AMIIDB extractive medium can effectively extract polar BAs through multi-interaction such as hydrophobic, π-π, hydrogen-bonding and dipole-dipole interactions because of the multiple functional groups in the sorbent.

Another preparation of a polymeric ionic liquid-based sorbent for SCSE was developed by Zhang et al. for the extraction of metal ions for the first time [42]. The SCSE sorbent was prepared in situ polymerization of 3-(1-ethyl imidazolium-3-yl) propyl-methacrylamido bromide and ethylene dimethacrylate and was used for the extraction of trace antimony in environmental water samples and combined with hydride generation atomic fluorescence spectrometry (HG-AFS) for the determination of trace antimony. The developed method has some advantages such as convenience, sensitivity, good reproducibility and cost-effectiveness, satisfactory linearity, and high recoveries.

In another study, Chen and Huang prepared a polymeric ionic liquid-based adsorbent as the extraction medium of stir cake sorptive extraction (SCSE) of three organic acid preservatives, namely, *p*-hydroxybenzoic acid, sorbic acid and cinnamic acid [43]. The synthesis of the adsorbent was carried out by the copolymerization of 1-ally-3-vinylimidazolium chloride (AV) and divinylbenzene (DVB) in the presence of a porogen solvent containing 1-propanol and 1,4-butanediol. After a long study to obtain the optimized conditions, the SCSE/AVDVB could extract the preservatives effectively through multiply interactions. In this framework, a simple and sensitive method by combining SCSE/AVDVB and HPLC/DAD was developed for the simultaneous analysis of the target preservatives in orange juices and tea drinks. The proposed method showed high sensitivity, good reproducibility, high cost-effectiveness and environmental friendliness.

Apart from the application of SPME for the determination of estrogens, SCSE has been used an alternative for the determination of estrogens in water samples was developed by Chen and coworkers [44]. In the framework of their study, a new PIL-based, a poly (1-ally-3-vinylimidazolium chloride-co-ethylene dimethacrylate)-AVED, monolith cake was prepared and used as sorbent of SCSE to extract trace estrogens with multi-interactions such as hydrophobic, π-π, hydrogen-bonding and dipole-dipole interactions effectively before their injection in HPLC-DAD system. The developed AVED/SCSE-LD-HPLC/DAD method showed a wide linear range, low LODs, satisfactory reproducibility and good recoveries for real water samples.

## 3. Conclusions

Over the last decade, many researchers have been attracted from the versatile character of ILs and PILs and their potentials. As discussed above, one of the most challenging applications of ILs is their usage in microextraction techniques as extractants, intermediate solvents, mediators, and desorption solvents. Table 2 summarizes the superior properties and characteristics of ILs in comparison with the common sorbent materials that are used in the miniaturized sample preparation techniques described in the present review [4,17,18,19,20,21,22,23,24,25,26,27,28,29,30,31,32,33,34,35,36,37,38,39,40,41,42,43,44]. As the IL-based microextraction techniques are budding gradually, some limitations should be noted, such as the high cost of their synthesis, their incompatibility with GC due to their low volatility and their potential toxicity. In general terms, the research in the field of ionic liquids will not stop evolving as the need for green analytical procedures is the priority of sample preparation. Taking into consideration their promiscuous properties and advantages, not only will the microextraction processed be improved, but also separation techniques, such as liquid and gas chromatography or electrophoresis, will expand their potentials.

## Figures and Tables

**Table 1:** Solvent selection guide.

Preferred	Usable	Undesirable
Water	Cyclohexane	Pentane
Acetone Ethanol	Heptane	Hexane(s)
2-Propanol	Toluene	Di-isopropyl ether
1-Propanol	Methylcyclohexane	Diethyl ether
Ethyl acetate	Methyl t-butyl ether	Dichloromethane
Isopropyl acetate	Isooctane	Dichloroethane
Methanol	Acetonitrile	Chloroform
Methyl ethyl ketone	2-MethylTHF	Dimethyl formamide
1-Butanol	Tetrahydrofuran	N-Methylpyrrolidinone
t-Butanol	Xylenes	Pyridine
Ils (nontoxic combinations of ions)	Dimethyl sulfoxide	Dimethyl acetate
	Acetic acid	Dioxane
	Ethylene glycol	Dimethoxyethane
		Benzene
		Carbon tetrachloride

**Table 2:** Limitations of the conventional sorbents and superior properties of ILs when they are used in the most commonly used miniaturized microextraction techniques.

SPME		DLLME		SBSE		SDME		SCSE	
Conventional Sorbents (PDMS, DVB, CAR, PEG, CW)	ILs	Conventional Organic Sorbents	ILs	Conventional Sorbents (PDMS, EG-silicone, PA)	ILs	Conventional Organic Sorbents	ILs	Conventional Sorbents (MIPs)	ILs
- low thermal stability of the fiber - short expiry date - small selectivity - fragility - limited operating time - decomposition during heating	- high thermal stability - high boiling points - no decomposition with heating	- low density - low viscosity and evaporation results to high instability of the drop	- high density allows phase separation - hydrophobic or hydrophilic nature - miscible or immiscible with the disperser solvent - high viscosity and surface tension allows to form larger, more stable droplets	- limited extraction efficiency towards polar and less polar compounds - limited number of available extraction solvents - physical damage to the extraction phase when stirring at high speed	- thermal stability - chemical stability	- low viscosity and evaporation results to high instability of the drop and poor precision levels	- high viscosity (formation of a larger-volume drop) - low vapor pressure - good thermal stability without evaporation - immiscibility with water	- low thermal stability - limited number of available extraction solvents	- mechanical stability - improved processability - durability - spatial controllability

PDMS: Polydimethylsiloxane, DVB: divinylbenzene, CAR: carboxen, PEG: polyethylene glycol, CW: Carbowax, EG: ethylene glycol, PA: polyacrylate.
